# New bacitracin-resistant nisin-producing strain of *Lactococcus lactis* and its physiological characterization

**DOI:** 10.3934/microbiol.2018.4.608

**Published:** 2018-10-11

**Authors:** Vakhtang V. Dzhavakhiya, Elena V. Glagoleva, Veronika V. Savelyeva, Natalia V. Statsyuk, Maksim I. Kartashov, Tatiana M. Voinova, Alla V. Sergeeva

**Affiliations:** 1 Laboratory of Biotechnology of Physiologically Active Compounds, Federal Research Centre “Fundamentals of Biotechnology”, Russian Academy of Sciences, Moscow 117312, Russia; 2 Department of Molecular Biology, All-Russian Research Institute of Phytopathology, Bolshie Vyazemy, Moscow region 143050, Russia; 3 FermLab LLC, Moscow 123592, Russia

**Keywords:** *Lactococcus lactis*, bacteriocins, nisin A, bacitracin, microbial drug resistance, oxidative stress

## Abstract

Nisin A belonging to the class I bacteriocins and produced by *Lactococcus lactis* subsp. *lactis* is widely used in many countries as highly efficient and safe preservative preventing growth of undesirable bacteria in food products. Though this compound is efficient at very low concentrations, reduction of its manufacturing cost is still relevant problem. An increased nisin A production requires improved resistance of its producer to nisin. According to some studies, mechanisms of microbial resistance to nisin A and bacitracin have a similar basis, and the same transporters are used to export these antibiotics from cells. To obtain strains with improved growth rate and nisin A productivity, selection of spontaneous bacitracin-resistant *L. lactis* mutants followed by examination of their stability as well as physiological and fermentation characteristics was carried out. Spontaneous mutants were obtained by culturing of *L. lactis* VKPM B-2092 strain on selective bacitracin-containing agar medium. The obtained bacitracin-resistant strain FL-75 was characterized by accelerated growth rate, doubled biomass accumulation, and improved nisin A resistance. The nisin A productivity of FL-75 exceeded that of the parental strain by 25% reaching 8902 U/mL after 14-h cultivation. In addition, FL-75 was characterized by the improved resistance to oxidative stress that has never been reported earlier for bacitracin-resistant microorganisms. Based on the performed characterization of FL-75, we can consider it as a new independent strain promising for the industrial production of food and feed biopreservatives. Comparison of published data and the obtained results allowed us to suppose that the bacitracin resistance mutation in FL-75 is determined rather by an increased expression of a gene homologous to the *bcrC* gene of *Bacillus* sp. than by the activation of multidrug resistance mechanisms. The revealed resistance of FL-75 to bacitracin and oxidative stress can be regulated by a common transcription factor activating in response to various environmental stresses.

## 1. Introduction

Many lactic acid bacteria possess antimicrobial activity caused by the production of bacteriocins [1]. Bacteriocins represent heterogeneous group of proteins consisting of both small peptides and low-molecular proteins with the molecular mass up to 30 kDa. Nisin A belongs to the subgroup of lantibiotics, low-molecular bacteriocins containing lanthionine and methyl lanthionine, which are produced by the binding of rare amino acids (dehydrobutyrine or dehydroalanine) with cysteine residues via thioether bridges [2]. Due to such structure, lantibiotics provide high antimicrobial activity even at nanomolar concentrations [3].

Lantibiotics are highly active towards Gram-positive bacteria. One of the most studied mechanisms of their antibacterial action is their binding with lipid II that inhibits biosynthesis of cell wall peptidoglycans and forms poration complexes in the cytoplasmic membrane resulting in cell death [4]–[6]. Nisin A is also able to inhibit some Gram-negative bacteria in the case of their previous treatment with chelators, such as EDTA, or pyrophosphate, which are able to increase the permeability of the thick cell wall of these bacteria [7],[8].

Since lantibiotics are produced only by Gram-positive bacteria [2] and are active mostly against the same type of bacteria, their microbial producers should protect themselves against their own metabolites. Thus, lantibiotic-producing strains have some specific protection mechanisms (so-called immunity system). In the case of nisin and subtilin, which have similar mechanisms of expression of specific proteins in lactic acid bacteria, this immunity is based on the expression of two proteins, LanI (membrane-associated lipoprotein) and LanFEG (ABC transporter localized in the cell membrane) [9],[10]. Interestingly that, though lantibiotics are differed in their size and specific activities, the systems of lantibiotic-specific immunity seem to be conserved for all species [11].

Nisin-producing strains protect themselves against nisin by the expression of two protein systems, NisFEG and NisI. NisFEG has been shown to expel nisin from the cell membrane [12]. NisI, a 27.8-kDa peptide consisting of 245 amino acid residues, inhibits the pore formation activity of nisin [2],[13]. Nevertheless, even such specific immunity does not provide a complete protection of microorganisms against the produced nisin: high concentrations of external nisin inhibit the growth of cells and may even kill them [13]. This fact determines the self-inhibiting effect of high nisin concentrations in nutrient medium. Microorganisms produce nisin until reaching of a certain host-specific “ceiling” level, when nisin production is stopped, even if the strain continues to grow [14]. This level is determined by the presence of a sufficient amount of nutrients and also by the nisin resistance of a microorganism.

Nisin A is widely used in many countries as highly efficient and safe preservative preventing bacteriological contamination of food products [15]–[17]. Though this antibiotic is efficient at very low concentrations, reduction of its manufacturing cost still remains to be relevant problem. Therefore, the study of the nisin resistance mechanisms in *L. lactis* and selection of more productive strains are of great commercial interest.

A large number of studies were intended to increase nisin production. One of the possible ways to improve nisin production is the optimization of fermentation conditions for nisin producers, which is able to provide a 2–4 fold increase in the nisin yield [18]–[21]. Another way is the development of genetically engineered strains with improved nisin resistance [22]. For example, increase in the number of the *nisFEG* gene copies in the *L. lactis* LL27 strain resulted in a 24% increase in the nisin production [23]. Introduction of a plasmid containing *nisZ*, *nisRK*, and *nisFEG* genes, involved into a nisin Z resistance and regulation of its biosynthesis, into *L. lactis* subsp. *lactis* A164 strain improved its nisin resistance and increased production of this bacteriocin [24]. However, the possibility of industrial application of bioengineered strains significantly depends on their stability, which can be negatively influenced by inactivating mutations occurring within a DNA region containing introduced genetic material, and also by horizontal gene transfer via mobile genetic elements, such as transposons and plasmids [25]–[27]. A possible loss of promoters or terminators within alien gene clusters is able to drastically influence on the possibility of the further industrial use of a strain. In addition, use of genetically modified organisms is often limited by both consumer acceptance issues and the necessity to get any regulatory approval for their use. Moreover, legislation of many countries directly prohibits the use of GM organisms in the food and drug production.

One of the known approaches to improve the export of target products from a cell is selection of a producer based on its resistance to a compound, which structure is similar to the target product and which is exported from cells using a similar transport system. In this connection, our attention was focused on the study showed that (1) export of bacitracin, a low-molecular antibiotic produced by *Bacillus licheniformis*, is performed via the ABC transporter system, and (2) amplification of genes encoding this transporter improves bacitracin biosynthesis [28]. Authors of another study identified a two-component BraS/BraR system essential for bacitracin and nisin A resistance in a pathogenic Staphylococcus aureus culture and showed the similarity between the mechanisms of bacitracin and nisin A resistance [29]. According to the results of that study, low bacitracin concentrations activate transcription of braDE and vraDE operons encoding ABC transporters, which play an important role in both nisin A and bacitracin resistance. Therefore, we hypothesized that the selection of *L. lactis* for bacitracin resistance may allow us to obtain strains resistant to higher nisin concentrations and, therefore, able to improve nisin A production.

The purpose of this study was the selection of spontaneous mutant *L. lactis* subsp. *lactis* strains highly resistant to bacitracin and characterized by improved growth rate and nisin A production level and the elucidation of possible mechanisms of such resistance.

## 2. Materials and methods

### 2.1. Microorganisms, reagents and fermentation conditions

Paper discs with antibiotics were purchased at the Saint-Petersburg Pasteur Institute (St.-Petersburg, Russia). Bacitracin was manufactured by Applychem (Darmstadt, Germany). Nisin A was manufactured by Chr. Hansen Co. (Hoersholm, Denmark). Alcalase was manufactured by Novozymes (Bagsvaerd, Denmark). All other reagents and ready culture media were manufactured by Fluka (Buchs, Switzerland). Skimmed milk and cheese whey were purchased at the Lianozovo milk processing factory of the Wimm-Bill-Dann JSC (Moscow, Russia).

The nisin-producing *L. lactis* subsp. *lactis* VKPM B-2092 strain obtained from the All-Russian Collection of Industrial Microorganisms (Moscow, Russia) was used as the parental strain. The spontaneous mutant strain FL-75 was selected from VKPM B-2092 as described below. Both strains were stored at −80 °С in MRS broth supplemented with 20% glycerine.

Fermentation medium contained the following components (g/L): skim milk powder, 30.0; cheese whey powder, 40.0; alcalase, 1.0; CaCO_3_, 50.0 (pH 7.0–7.2).

Stock cultures were grown for 24 h in skimmed milk at 30 °С under temperature-controlled conditions and then transferred into 250-mL flasks containing 50 mL of fermentation medium or into a 4-L pH-controlled Biotron bioreactor (Incheon, South Korea); the inoculate volume was 10% of the fermentation medium volume. Fermentation was carried out for 19–20 h at 30 °С. In the case of flasks, it was carried out at 220 rpm using a RC-TK Infors rotation shaker (Einsbach, Germany).

The pH values for nutrient media and reagent solutions were determined potentiometrically using a Seven Easy pH meter (Mettler-Toledo, Giesen, Germany). pH control of culture broth in the bioreactor was carried out using a sterilized pH sensor (Mettler Toledo, Giesen, Germany).

### 2.2. Selection of bacitracin-resistant mutant strains

Culture of VKPM B-2092 grown in skimmed milk for 24 h under stationary conditions (30 °С, no aeration) was used as initial material. The selection was carried out using M17 agar medium supplemented with increasing bacitracin concentrations (4–16 µg/mL). Strains showed good growth in Petri plates with selective medium were repeatedly passaged via monoclonal inoculation with the assessment of their bacitracin resistance and then tested for nisin A productivity by the agar diffusion method [30].

### 2.3. Strain stability assessment

The stability of inheritance of the increased nisin A productivity of the most promising strains was assessed by ten successive subculturings accompanied by the assessment of the nisin A activity level for each subculturing. At the final stage, several selected strains were also assessed for the variability of their nisin A activity level among clones of the same population to select strains with the lowest variability.

### 2.4. Physiological characterization of strains

The obtained mutant FL-75 strain was compared with VKPM B-2092 in several physiological characteristics, such as antibiotic and ethidium bromide resistance, oxidative stress resistance, and also some fermentation characteristics including cell growth rate, nisin A activity level, lactose consumption, and lactate accumulation (assessed via the NaOH consumption). A comparison of fermentation characteristics was carried out using averaged results of two fermentations per each strain arranged in the 4-L bioreactor; in this case, fermentation medium was supplemented with additional glucose (0.5%).

To evaluate antibiotic resistance, 0.1 mL (1 × 10^9^ cells) of each one-day culture was inoculated onto MRS agar medium poured into Petri plates. Paper discs pre-impregnated with standard concentrations of antibiotics (kanamycin, 30 µg; gentamycin, 10 µg; tobramycin, 10 µg; streptomycin, 10 µg; tunicamycin, 10 µg; tetracycline, 30 µg; vancomycin, 30 µg; lincomycin, 15 µg; and chloramphenicol, 30 µg) were applied onto the surface of agar plates. Cultures were incubated for 48 h at 30 °С, and then bacterial growth around each disc was evaluated. Antibiotic sensitivity of each culture was determined by the size of growth inhibition zones.

To evaluate sensitivity of strains to ethidium bromide, a range of dilutions (from 10^−2^ to 10^−8^) of one-day cultures was prepared, and 0.1 mL of each dilution was inoculated onto MRS agar with or without ethidium bromide (20 µg/mL). Petri plates were incubated for 48 h at 30 °С, and then the sensitivity level of each strain was accessed by the number of grown colonies. Evaluation of the strain sensitivity to bacitracin and nisin A was carried out in the same manner; the tested antibiotic concentrations were 10 and 20 µg/mL (bacitracin) or 50 and 100 µg/mL (nisin A).

Lactose content was determined by the Bertrand's method [27]. Lactic acid content was evaluated by titration with 10% NaOH [27]. Concentration of viable cells (CFU/mL) was determined by inoculation of corresponding sample dilutions onto MRS agar [31].

Evaluation of the proteolytic activity of strains was performed using the method described in [32] with some modifications. The method is based on a formation of white precipitation zones in casein-containing agar medium due to proteolytic activity of microbial enzymes. The medium used for this assay had the following composition (%): agar, 1.4; sodium caseinate, 1; MgCl_2_, 0.1 (pH 6.2–6.5). Agar was melted on a water bath, supplemented with the rest of components, and poured into Petri dishes (2-mm layer) without sterilization. After solidification, 6-mm wells were cut in the agar, and 0.05 mL of the suspended cells of the examined strain was added into each well. After a 2-h incubation at 30 °C, the presence of white zones around the wells was examined.

### 2.5. Statistical treatment

Statistical treatment of obtained data was carried out via calculation of the relative reproducibility dispersion and the confidence interval for the mean value [33]. The relative reproducibility dispersion was calculated for each measured index and averaged over the total data array obtained for all experiments (3 or 5 repetitions for each measured parameter). Calculations were carried out using a Statistica 6.0 software (StatSoft Inc., Tulsa, USA).

## 3. Results

### 3.1. Selection of bacitracin-resistant mutant strains with improved nisin A productivity

**Figure 1.**
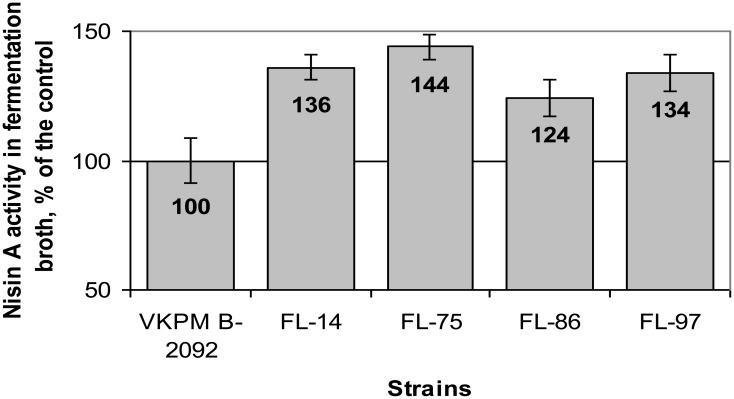
Nisin A biosynthesis level in initial (VKPM B-2092) and selected mutant strains of *Lactococcus lactis* subsp. *lactis*. The VKP B-2092 strain was used as a control.

The growth of the initial VKPM B-2092 strain on the selective medium was completely suppressed at the bacitracin concentration of 4 µg/mL. During selection process, spontaneous mutants grown on the medium containing 16 µg/mL of bacitracin demonstrated weak growth and low ability to produce nisin, so they were excluded from the further consideration. The number of mutant strains resistant to 4–14 µg/mL of bacitracin reached 100. After examination of their nisin production level comparing to that of VKPM B-2092 and evaluation of the stability of this parameter for at least five successive generations, four best strains were selected. The selective bacitracin concentrations for these strains were 14 (FL-97), 10 (FL-14), and 8 (FL-75, FL-86) µg/mL. Nisin productivity of selected strains is shown in Figure 1; the most productive strain was FL-75 (144% of the VKPM B-2092 productivity).

### 3.2. Strain stability assessment

**Figure 2.**
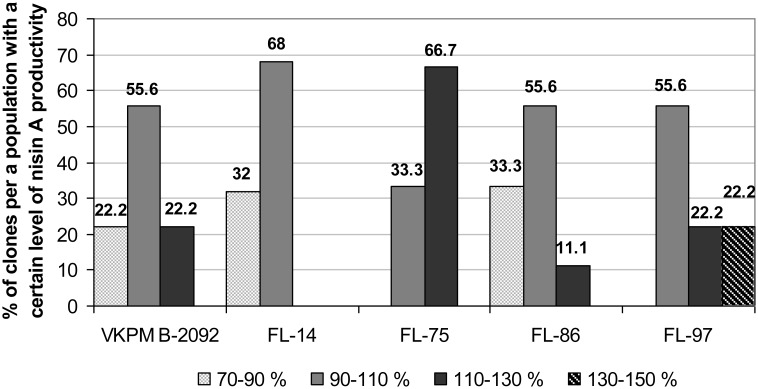
Variability of spontaneous mutants of *Lactococcus lactis* subsp. *lactis* concerning the nisin A productivity as compared to that of the parental VKPM B-2092 strain.

Due to performed selection, the nisin yield of the selected four strains was significantly higher than that of the parental strain. Since the stability of a target product biosynthesis is very important for industrial microorganisms, strains were examined for the variability of their nisin A activity level among different clones of the same population. Results of this examination are shown in Figure 2. For each strain, the percentage of clones, demonstrating four different levels of nisin A activity comparing to the activity of the parental strain at the beginning of the experiment (control), was calculated to determine strains, for which the majority of clones showed improved nisin A production. The most stable results concerning the maintenance of clones with the improved productivity in the population were observed for FL-75 and FL-97, which were characterized by the absence of clones with the nisin A activity below control (column “70–90%”). Since FL-75 demonstrated the highest nisin A productivity (Figure 1), it was chosen for the further study.

### 3.3. Comparison of fermentation characteristics of VKPM B-2092 and FL-75

**Figure 3.**
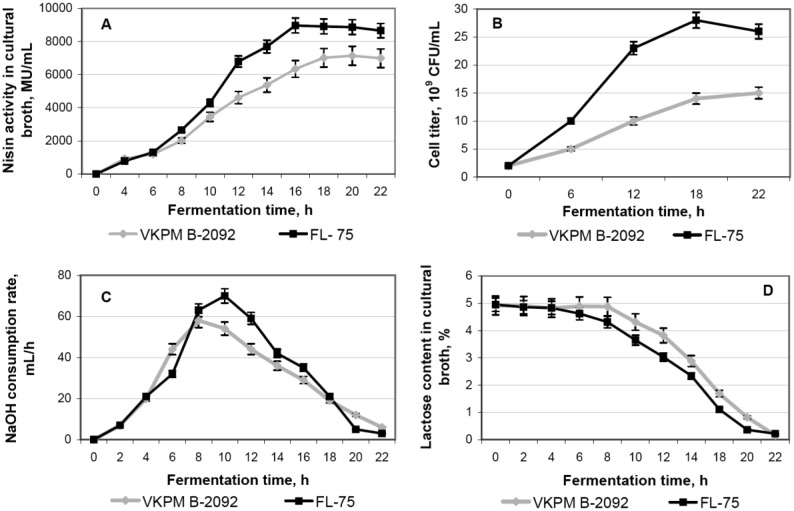
Fermentation characteristics of parental (VKPM B-2092) and mutant (FL-75) strains of *Lactococcus lactis* subsp. *lactis*. A, nisin A activity in fermentation broth; B, cell growth rate; C, consumption rate of 10% NaOH; D, lactose consumption rate.

Dynamics of changes of the examined fermentation parameters is shown in Figure 3A–D. In the case of FL-75, the maximum nisin A production was reached 16 h after the start of fermentation and remained at this level for 4 h, whereas the maximum productivity level of the parental strain was achieved only after 20 h of fermentation (Figure 3A). The growth rate of FL-75 significantly exceeded that of VKPM B-2092; to the end of fermentation, the concentration of viable cells was almost twice more than that in VKPM B-2092 (Figure 3B). The lactose and NaOH consumption (i.e., lactate accumulation) rates of the mutant FL-75 strain exceeded those of the parental strain in the period between 8 and 16 h of fermentation (Figure 3C,D).

Using data on the concentration of viable cells and the total nisin A output per unit of volume, the specific productivity of both cultures during the fermentation was calculated (Table 1). Unlike total productivity, the specific productivity of FL-75 was lower that that of VKPM B-2092 that can be explained by a twice higher biomass accumulated to the end of fermentation. Since the proteolytic activity of cells did not differ between both strains, the lower specific productivity of FL-75 is probably caused by either limitation of exogenous amino acids, involved into nisin A biosynthesis (because of their higher consumption for the biomass accumulation), or the nonoptimal concentration of a protein substrate in the nutrient medium. Therefore, the further medium improvement for the mutant strain should be carried out.

**Table 1.** Specific nisin A productivity of parental (VKPM B-2092) and mutant (FL-75) strains of *Lactococcus lactis* subsp. *lactis* during fermentation.

Fermentation time, h	Nisin A activity, U/mL			Concentration of viable cells, 10^9^ CFU/mL			Specific nisin A activity, U/10^9^	
		
	VKPM B-2092	FL-75		VKPM B-2092	FL-75		VKPM B-2092	FL-75
4	1195	1306		3	10		398	131
6	4607	6784		10	23		461	421
14	7017	8902		14	28		504	423
22	6877	8652		16	26		430	333

### 3.4. Comparison of antibiotic resistance of VKPM B-2092 and FL-75

According to the obtained results, no significant difference was observed between VKPM B-2092 and FL-75 concerning their resistance to the tested antibiotics (Table 2) and ethidium bromide (Table 3) except of tetracycline and lincomycin. At the same time, FL-75 demonstrated an improved resistance to bacitracin and nisin A (Table 3).

**Table 2.** Antibiotic resistance of *Lactococcus lactis* subsp. *lactis* VKPM D-2092 and FL-75 strains.

Antibiotic	Growth inhibition zone, mm	
	VKPM B-2092	FL-75
Kanamycin	17.1 ± 1.2	18.2 ± 1.1
Gentamycin	15.0 ± 1.1	17.2 ± 1.3
Tobramycin	28.0 ± 1.2	25.1 ± 1.2
Streptomycin	18.3 ± 1.0	19.1 ± 0.9
Tunicamycin	15.1 ± 1.0	14.0 ± 0.8
Tetracycline	28.1 ± 1.5	22.2 ± 1.3
Vancomycin	20.2 ± 1.2	18.9 ± 1.1
Lincomycin	19.0 ± 1.3	24.0 ± 1.4
Chloramphenicol	31.1 ± 1.5	29.9 ± 1.4

**Table 3.** Resistance of *Lactococcus lactis* subsp. *lactis* VKPM D-2092 and FL-75 to ethidium bromide (EB), nisin A, and bacitracin.

Solid medium variant	Number of viable cells, CFU/mL	
	VKPM B-2092	FL-75
EB-free (Control)	4 × 10^9^	5 × 10^9^
EB (20 µg/mL)	2 × 10^2^	2 ×10^2^
Nisin-free (Control)	6 × 10^9^	6 × 10^9^
Nisin (50 µg/mL or 2000 MU/mL)	3 × 10^9^	4 × 10^9^
Nisin (100 µg/mL or 4000 MU/mL)	2 × 10^9^	4 × 10^9^
Bacitracin-free (Control)	4 × 10^9^	4 × 10^9^
Bacitracin (10 µg/mL)	zero growth	3 × 10^9^
Bacitracin (20 µg/mL)	zero growth	2 × 10^9^

### 3.5. Oxidative stress resistance

**Figure 4.**
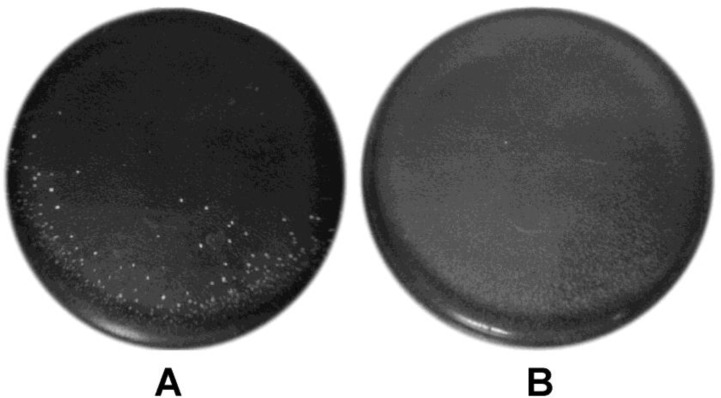
Effect of oxidative stress caused by a 5 mM H_2_O_2_ addition to nutrient medium on the growth of *Lactococcus lactis* subsp. *lactis* VKPM B-2092 (A) and FL-75 (B).

An interesting distinctive feature of FL-75 was its resistance to oxidative stress caused by the supplementation of solid nutrient medium with 5 mM H_2_O_2_. The initial inoculum concentration was 10^8^ CFU/mL. Inoculation of FL-75 on such medium resulted in a lawn formation, whereas VKPM B-2092 formed single colonies (Figure 4). The number of viable cells for FL-75 and VKPM B-2092 was ∼5 × 10^8^ and 2 × 10^3^ CFU/mL, respectively.

Thus, the revealed difference between the parental and mutant strains concerning the examined characteristics makes it possible to consider FL-75 to be a stable independent strain. This strain was deposited to the All-Russian Collection of Microorganisms (Pushchino, Moscow region) under the accession number VKM B-2056D.

## 4. Discussion

To date, there are two known basic mechanisms of bacterial resistance to bacitracin. The first one is related to the export of this antibiotic from cells by a BcrABC transporter [28],[34], another one involves the enhanced biosynthesis of C55-isoprenyl phosphate, a precursor of peptidoglycans [35], by BacA undecaprenol kinase [36].

BcrABC transporter provides bacitracin resistance of bacitracin-producing *B. licheniformis*. BcrB and BcrC proteins form a transmembrane channel, whereas two BcrA molecules work as ATPases providing energy required for the process of bacitracin transportation [34]. Homologues of *bcrABC* genes from *B. licheniformis* were also revealed in *B. subtilis* [37]. The study of spontaneous bacitracin-resistant strains of *B. subtilis* showed this resistance type is connected with the product of the *ywoA* gene homologous to the *bcrC* gene. Disruption of *ywoA* gene caused hypersensitivity of mutant strains to bacitracin, but did not influence on their sensitivity to other drugs such as surfactin, iturin A, vancomycin, tunicamycin, ethidium bromide, and deoxycholate. These data indicate that the bacitracin resistance can be specifically provided by the ywoA protein (BcrC) without any involvement of multidrug resistance (MDR) transporters [34],[37]. According to the existing data, *L. lactis* has several MDR-type transporters, such as YdaG, YdbA, and LmrA; they are involved into the export of various toxic compounds including antibiotics, ethidium bromide, and bacteriocins [38].

Comparison of published data and results of this study makes it possible to suppose that the bacitracin resistance mutation in FL-75 may be determined rather by increased expression of a gene homologous to the *bcrC* gene of *Bacillus* sp. than by activation of MDR mechanisms. Like in the case of bacitracin-resistant mutants of *B. subtilis* [37], FL-75 remains sensitive to ethidium bromide, tunicamycin, vancomycin, and some other antibiotics.

An increased nisin A resistance of *L. lactis* was shown to be accompanied by increased expression of bacitracin resistance operon (ysaBCD) genes [39]. Paradoxically, the *L. lactis* Nis^R^ strain mentioned in that study was even more sensitive to bacitracin than the parental strain. This fact was probably caused by the lack of any enhancement of the expression of the *bacA* gene, which product provides cell protection against the bacitracin-caused inhibition of a C55-isoprenyl phosphate biosynthesis critical for the cell wall formation [40].

Improved physiological characteristics of FL-75, which has better nisin A resistance than the parental strain, are probably related to the increased expression of both *bacA* and *ysaBC* (a *bcrC* homologue) genes, which, due to the accelerated bacitracin release, attenuates the toxic impact of nisin A on the cell growth and carbohydrate metabolism. As a result, the total productivity of FL-75 exceeds that of the parental strain by 20–25% because of a significant increase in the nisin A-producing biomass.

Another aspect of the bacitracin resistance mutation is related to the increased oxidative stress resistance of FL-75. As far as we know, this resistance type has never been reported for bacitracin-resistant microorganisms. The similar phenomenon was observed in the *L. lactis* Nis^R^ strain: increased nisin A resistance was accompanied by the overexpression of *ynhCD* genes encoding the tellurite resistance protein [39]. The toxicity of oxyanione tellurite TeO_3_^2−^ is connected with the oxidation of thyol groups of glutathione [41], which protects *L. lactis* cells from the oxidative and acid stress [42],[43].

Some authors note a correlation between the resistance to various stress factors and the redox potential of *L. lactis* cells. For example, rapid adaptation of L. lactis subsp. cremoris MG1363 to the oxygen stress was reported at the transcriptional and metabolic levels in the case of initially high oxygen content in nutrient medium; this adaptation provided normal medium acidulation and reduction of the redox potential of the medium [44]. Obviously, the revealed resistance of FL-75 to bacitracin and oxidative stress is regulated by a common transcription factor, which is activated in response to various environmental stresses. A more detailed study of the mechanisms of resistance to the above-mentioned factors requires additional biochemical and molecular investigations including the transcriptomic analysis. We plan to arrange such investigation as a separate study.

## 5. Conclusions

Due to the selection of *L. lactis* subsp. *lactis* for the bacitracin resistance, we obtained a spontaneous mutant strain FL-75 characterized by improved nisin A productivity and growth characteristics (improved growth rate and biomass accumulation as well as the accelerated lactose consumption and lactate accumulation). In addition, FL-75 is distinguished by improved oxidative stress resistance, a phenomenon that has never been reported earlier for bacitracin-resistant microorganisms. The new strain seems to be very promising for the industrial production of food and feed biopreservatives.
